# Spin-helix Larmor mode

**DOI:** 10.1038/s41598-018-21818-8

**Published:** 2018-02-22

**Authors:** Shahrzad Karimi, Carsten A. Ullrich, Irene D’Amico, Florent Perez

**Affiliations:** 1 Department of Physics and Astronomy, University of Missouri, Columbia, MO 65211 USA; 2 Department of Physics, University of York, York, YO10 5DD United Kingdom; 3 Institut des Nanosciences de Paris, CNRS/Université Paris VI, Paris, 75005 France

## Abstract

A two-dimensional electron gas (2DEG) with equal-strength Rashba and Dresselhaus spin-orbit coupling sustains persistent helical spin-wave states, which have remarkably long lifetimes. In the presence of an in-plane magnetic field, there exist single-particle excitations that have the character of propagating helical spin waves. For magnon-like collective excitations, the spin-helix texture reemerges as a robust feature, giving rise to a decoupling of spin-orbit and electronic many-body effects. We prove that the resulting spin-flip wave dispersion is the same as in a magnetized 2DEG without spin-orbit coupling, apart from a shift by the spin-helix wave vector. The precessional mode about the persistent spin-helix state is shown to have an energy given by the bare Zeeman splitting, in analogy with Larmor’s theorem. We also discuss ways to observe the spin-helix Larmor mode experimentally.

## Introduction

Spin-flip waves are collective excitations of magnetic systems^1,2^: rather than flipping individual magnetic moments, which causes a large exchange energy penalty, the periodic reversal of magnetic moments extends as a precessional wave over the entire system, which is energetically favorable. There has been recent interest in spin waves in ferromagnetic thin films as an information carrier, which constitutes the basis for magnonics^3,4^. Spin waves exist in systems with localized and itinerant magnetic moments. In the latter case, the precession of the interacting spins, the charge motion, and the spin-orbit coupling (SOC) due to inversion asymmetry are all interrelated and lead to novel phenomena. For example, chiral spin waves have been observed in asymmetric monolayers of iron^5,6^ and on the surface of topological insulators^7^, helical spin waves have been predicted in a two-dimensional electron gas (2DEG) subject to Rashba SOC^8^, and twisted spin waves have been predicted and observed in magnetized 2DEGs^9^.

The fundamental and practical aspects of spin waves in the presence of SOC have drawn interest recently in the context of spintronics^10–13^. SOC provides the conversion of charge based information into the spin wave^14,15^. However, the presence of both SOC and Coulomb interaction still poses interesting challenges, especially in the dynamical regime.

In this paper, we will study spin waves in a 2DEG in the presence of in-plane magnetization and SOC. This system exhibits a rich interplay between Coulomb many-body effects, Rashba and Dresselhaus SOC, applied magnetic field, and electron density, which we have studied earlier^9,16–18^. What we found is that the spin waves are modified by the SOC in a subtle manner: the spin waves get a boost of their group velocity whose magnitude and orientation depends on the crystallographic propagation direction in the quantum well plane. This interesting behavior of the spin waves can be understood via a transformation into a spin-orbit twisted reference frame; however, in general this only holds to lowest order in SOC^9^.

In this paper, we consider a very special case in which exact results can be proved to all orders in SOC, namely, the case of a persistent spin helix^19–27^. The spin helix arises in a 2DEG in which the strengths of the Rashba and Dresselhaus fields, *α* and *β*, are equal (in this paper we only include contributions to the Dresselhaus effect which are linear in the wavevector, i.e., *β* = *β*_1_; the cubic *β*_3_ coupling coefficient^23,27^ is ignored). We here consider a 2DEG embedded in a zincblende quantum well grown along the [001] direction: SU(2) symmetry is then partially restored, and a helical spin texture can be sustained along the [110] direction. This result is robust against spin-independent disorder scattering and Coulomb interactions^20^. The main experimental signature of this state is that spin packet excitations are protected from decoherence, leading to extraordinarily long lifetimes^22,25^.

If a magnetic field is applied in the plane of a 2DEG with *α* = *β*, spin-packet excitations can sustain long-lived precessional motion^23^. Furthermore, some interesting magnetoelectronic effects can occur in 2DEGs^28–30^ or quantum wires^31,32^ which reflect the special condition *α* = *β*. However, to our knowledge the spin-wave dynamics under these circumstances, which involves Coulomb interactions between the electrons at finite magnetic field, has not been explicitly addressed before.

Our treatment of spin waves is based both on time-dependent density-functional theory (TDDFT) in the linear-response regime^33^ and on an equation-of-motion approach featuring the full many-body Hamiltonian^9,34^. We derive the exact form of the spin-wave dispersion for systems with a spin-helix texture, and find that it is obtained from the dispersion without SOC by a simple wave vector shift. The spin-wave stiffness *S*_s*w*_ remains unchanged.

The main result of this paper is that we identify an exact dynamical state of the 2DEG with *α* = *β*, which can be characterized as a collective precession of the 2DEG about the spin-helix state. The precession occurs at the bare Zeeman frequency, and we therefore refer to it as the spin-helix Larmor mode. This mode will be characterized by its long lifetime, and we will discuss ways in which it could be experimentally observed.

## Results

### 2DEG with spin-orbit coupling in a magnetic field: the spin helix

We consider the electronic ground state of a 2DEG in an *n*-doped zincblende quantum well in the presence of an in-plane magnetic field and Rashba and Dresselhaus SOC. Since the magnetic field is parallel to the 2DEG, it only acts on the spin and there is no Landau level quantization (as long as the magnetic length ${l}_{B}=\sqrt{\hslash /|eB|}$ is not significantly smaller than the well width). We will use the effective-mass approximation and work in units where $\hslash$ = e^*^ = m^*^ = 1, where e^*^ and m^*^ are the effective charge and mass, respectively.

**Figure 1:**
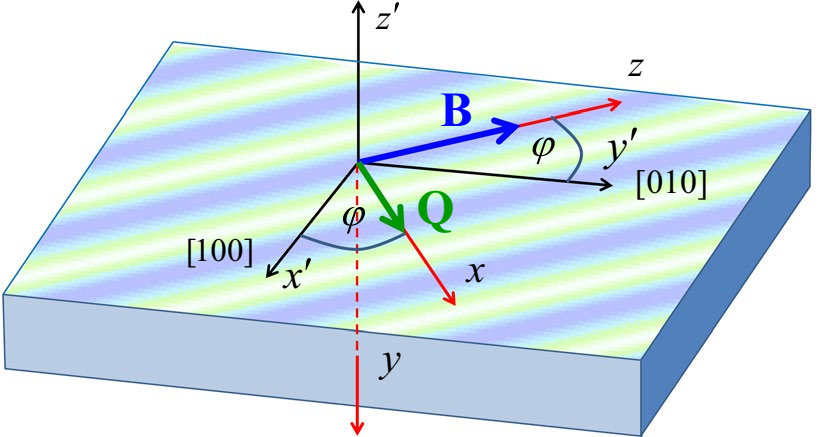
Reference frames ${\mathcal R} ^{\prime}$ (black) and ${\mathcal R}$ (red) for the electronic states in a 2DEG with SOC and in-plane magnetic field **B**. The striped pattern along the *φ* = 45° direction indicates the persistent spin helix state of the 2DEG, with wave vector **Q**, which forms in the absence of **B** if the Rashba and Dresselhaus coupling strengths are equal.

Figure 1 defines two reference frames. The primed frame ${\mathcal R} ^{\prime}$ is fixed with respect to the quantum well: the *x*′-, *y*′- and *z*′-axes point along the crystallographic [100], [010], and [001] directions, respectively; the 2DEG is in the *x*′ − *y*′ plane. The Rashba and Dresselhaus SOC fields will be introduced in ${\mathcal R} ^{\prime}$, but it will be convenient for the discussion of spin waves to work in a coordinate system ${\mathcal R}$ which is oriented such that its *x* and *z* axes lie in the quantum well plane, and the *z*-axis points along the in-plane magnetic field **B**. As shown in Fig. 1, the *x*-axis is at an angle *φ* with respect to the *x*′-axis.

### Single-particle states

Without magnetic field or SOC, the lowest electronic conduction subband in the quantum well is spin-degenerate. For simplicity, we will treat the electronic states as purely two-dimensional; however, the main results in this paper will not change qualitatively if one takes the finite well width into account. The magnetic field lifts the degeneracy and splits the lowest subband into two, which we shall denote by the index *p* = ±1. In the reference frame ${\mathcal R} ^{\prime}$, the associated single-particle states can be written as Φ′_*p***k**_(**r**′) = *e*^*i***k** ⋅ **r**′^Ψ′_*p***k**_, where **r**′ = (*x*′, *y*′), *k* = (*k*_*x*′_, *k*_*y*′_), and Ψ′_*p***k**_ is a two-component spinor of the form

1 $${{\boldsymbol{\Psi }}{\boldsymbol{^{\prime} }}}_{p{\bf{k}}}=(\begin{array}{c}{\psi ^{\prime} }_{p{\bf{k}}\uparrow }\\ {\psi ^{\prime} }_{p{\bf{k}}\downarrow }\end{array})\mathrm{.}$$

Here, “spin-up“ and “spin-down” (↑ and ↓) refer to the spin quantization axis *z*′.

The states Ψ′_*p***k**_ are obtained from the following Kohn-Sham single-particle equation:

2 $$(\frac{{k}^{2}}{2}{\hat{\sigma }}_{0}+{h}_{x^{\prime} }{\hat{\sigma }}_{x^{\prime} }+{h}_{y^{\prime} }{\hat{\sigma }}_{y^{\prime} }){{\boldsymbol{\Psi }}{\boldsymbol{^{\prime} }}}_{p{\bf{k}}}={E}_{p{\bf{k}}}{{\boldsymbol{\Psi }}{\boldsymbol{^{\prime} }}}_{p{\bf{k}}}\,,$$

where ${\hat{\sigma }}_{\mathrm{0,}x^{\prime} ,y^{\prime} ,z^{\prime} }$ are the usual Pauli matrices. The off-diagonal parts in Eq. (2) involve

3 $${h}_{x^{\prime} }=-\frac{Z+{Z}_{{\rm{x}}{\bf{c}}}}{2}\,\sin \,\phi +\alpha {k}_{y^{\prime} }+\beta {k}_{x^{\prime} }$$

4 $${h}_{y^{\prime} }=\frac{Z+{Z}_{{\rm{x}}{\bf{c}}}}{2}\,\cos \,\phi -\alpha {k}_{x^{\prime} }-\beta {k}_{y^{\prime} }\mathrm{.}$$

Here, *Z* = g^*^μ_*B*_*B* is the bare Zeeman energy (*μ*_*B*_ is the Bohr magneton, and g^*^ is the effective *g*-factor). The presence of Coulomb many-body effects in the interacting 2DEG gives rise to the Zeeman exchange-correlation (xc) energy *Z*_x**c**_, which we discuss below. *α* and *β* are the usual Rashba and Dresselhaus linear coupling parameters; we ignore contributions to the Dresselhaus effect that are cubic in the wavevector, since these tend to be much smaller than the linear contributions^23,27^.

It is convenient to change the reference frame for the spin, and go over to reference system ${\mathcal R}$, whose *z*-axis is along the magnetic field direction. We introduce two in-plane vectors, **q**_0_ and **q**_1_, given in ${\mathcal R} ^{\prime}$ by

5 $${{\bf{q}}}_{0}=(\alpha \,\cos \,\phi +\beta \,\sin \,\phi ){\hat{e}}_{x^{\prime} }+(\alpha \,\sin \,\phi +\beta \,\cos \,\phi ){\hat{e}}_{y^{\prime} }$$

6 $${{\bf{q}}}_{1}=(\beta \,\cos \,\phi -\alpha \,\sin \,\phi ){\hat{e}}_{x^{\prime} }+(\alpha \,\cos \,\phi -\beta \,\sin \,\phi ){\hat{e}}_{y^{\prime} }\mathrm{.}$$

With this, Eq. (2) transforms into

7 $$[\frac{{k}^{2}}{2}{\hat{\sigma }}_{0}+(\frac{Z+{Z}_{{\rm{x}}{\bf{c}}}}{2}-{\bf{k}}\cdot {{\bf{q}}}_{0}){\hat{\sigma }}_{z}+{\bf{k}}\cdot {{\bf{q}}}_{1}{\hat{\sigma }}_{x}]{{\rm{\Psi }}}_{p{\bf{k}}}={E}_{p{\bf{k}}}{{\rm{\Psi }}}_{p{\bf{k}}},$$

where the scalar products **k** ⋅ **q**_0_ and **k** ⋅ **q**_1_ remain invariant under ${\mathcal R} {^{\prime} }$ → ${\mathcal R}$. Ψ_*p***k**_ is now a two-component spinor whose spatial coordinates and spin quantization axes are defined with respect to ${\mathcal R}$.

Let us now discuss the xc contribution. The in-plane magnetic field causes the 2DEG to become uniformly magnetized. The xc energy per particle of a homogeneous 2DEG^35^, *e*_xc_(*n*, *ζ*), can be written as a functional of the density *n* and the spin polarization *ζ*, where *n* = *n*_↑_ + *n*_↓_ and *ζ* = (*n*_↑_ − *n*_↓_)/*n* (↑ and ↓ are now defined with respect to ${\hat{e}}_{z}$). The Zeeman xc energy is then given by

8 $${Z}_{{\rm{xc}}}\mathrm{=2}\frac{\partial {e}_{{\rm{x}}{\bf{c}}}}{\partial \zeta }\,\mathrm{.}$$

The renormalized Zeeman energy^36^ can now be defined as Z^*^ = *Z* + *Z*_xc_.

The general solution of Eq. (7) has been considered elsewhere^37^; instead, we concentrate here on the special case *α* = *β* and *φ* = *π*/4 or 5*π*/4. Under these circumstances, **q**_1_ = 0 and Eq. (7) simplifies considerably:

9 $$[\frac{{k}^{2}}{2}{\hat{\sigma }}_{0}+\frac{{Z}^{\ast }-{\bf{k}}\cdot {\bf{Q}}}{2}{\hat{\sigma }}_{z}]{{\rm{\Psi }}}_{p{\bf{k}}}={E}_{p{\bf{k}}}{{\rm{\Psi }}}_{p{\bf{k}}},$$

where

10 $${\bf{Q}}=\pm 4\alpha {\hat{e}}_{x},\quad \phi =\{\begin{array}{l}\pi \mathrm{/4}\\ 5\pi \mathrm{/4.}\end{array}$$

To keep the discussion a bit simpler, we will limit ourselves to the case *φ* = *π*/4 in the following, so ${\bf{Q}}\mathrm{=4}\alpha {\hat{e}}_{x}$ (the *φ* = 5*π*/4 case is essentially the same, just in the opposite direction).

The solution of Eq. (9) is straightforward. We obtain

11 $${{\rm{\Psi }}}_{+,{\bf{k}}}=(\begin{array}{c}1\\ 0\end{array}),\quad {{\rm{\Psi }}}_{-,{\bf{k}}}=(\begin{array}{c}0\\ 1\end{array}),$$

where

12 $${E}_{\pm ,{\bf{k}}}=\frac{{k}^{2}}{2}\pm \frac{{Z}^{\ast }-{\bf{k}}\cdot {\bf{Q}}}{2}\,\mathrm{.}$$

**Figure 2:**
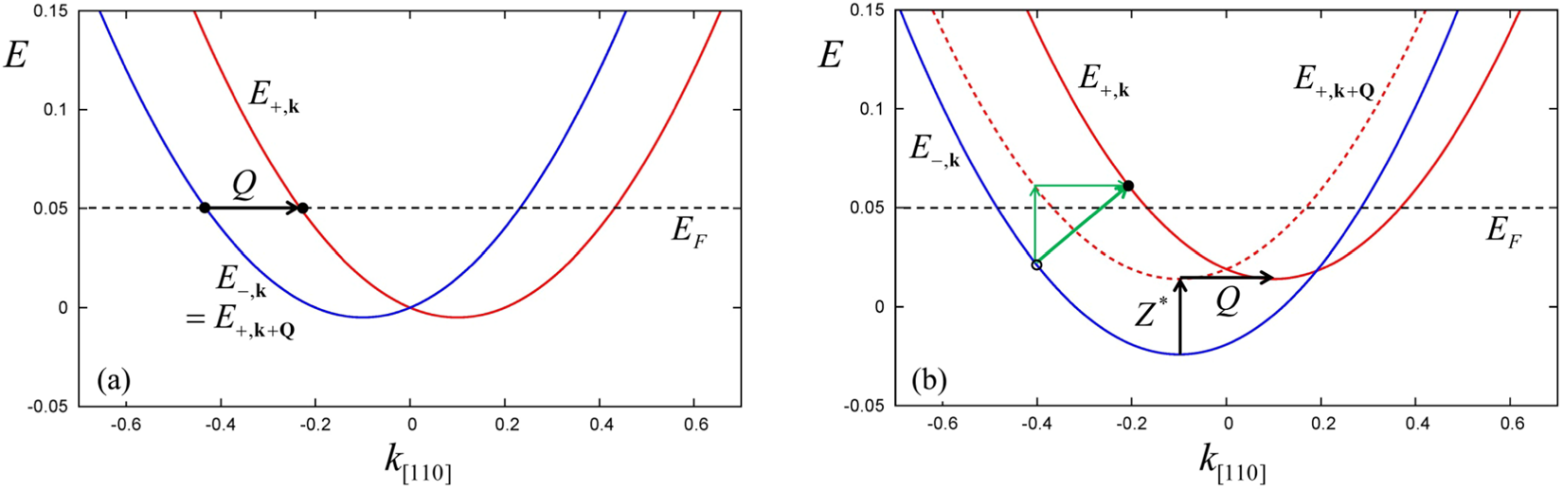
Single-particle energies *E*_−,**k**_ and *E*_+,**k**_ for *α* = 0.05 and **k** along the [110] direction, see Eq. (13). (**a**) No magnetic field (Z^*^ = 0). Linear combinations of *E*_−,**k**_ and *E*_+,**k**+**Q**_ states have spin helix texture, but these cancel out if summed over all occupied states below *E*_*F*_. A persistent spin helix appears if a quasiparticle is injected at the Fermi surface, as shown. (**b**) Finite magnetic field (Z^*^ = 0.0381). Single-particle excitations across the Fermi energy with momentum transfer **Q** (thick green arrow) give rise to propagating spin helices.

The single-particle energies (12) have the important property

13 $${E}_{+,{\bf{k}}+{\bf{Q}}}={E}_{-,{\bf{k}}}+{Z}^{\ast },$$

which is illustrated in Fig. 2b, using the parameters Z^*^ = 0.0381 and *α* = 0.05. This large value of *α* (typical experimental values of *α* are about an order of magnitude smaller) was chosen for clarity of presentation.

### B = 0: spin-helical single-particle eigenstates

In the absence of external magnetic fields, i.e., for Z^*^ = 0 (see Fig. 2a), the degeneracy of the two energy branches *E*_+,**k**+**Q**_ and *E*_−,**k**_ gives rise to a persistent spin-helix state of the 2DEG, as illustrated by the stripe-like pattern in Fig. 1. This is easy to see: Due to the degeneracy, linear combinations of the eigenstates Ψ_−,**k**_ and Ψ_+,**k**+**Q**_ are also solutions of Eq. (9). The single-particle wave functions for wave vector **k** can therefore be written as

14 $${{\rm{\Phi }}}_{{\bf{k}}}^{{\boldsymbol{\pm }}}({\bf{r}})=a(\begin{array}{c}1\\ 0\end{array}){e}^{i({\bf{k}}+{\bf{Q}})\cdot {\bf{r}}}\pm b(\begin{array}{c}0\\ 1\end{array}){e}^{i{\bf{k}}\cdot {\bf{r}}},$$

where |*a*|^2^ + |*b*|^2^ = 1. From the associated spin-density matrix it is straightforward to determine the magnetization in the ${\mathcal R}$ frame. We obtain *m*_*z*_ = |*a*|^2^ − |*b*|^2^, and since the macroscopic magnetization must vanish (i.e., *m*_*z*_ = 0), this implies |*a*| = |*b*|. Writing $a,b={e}^{i{\varphi }_{a,b}}/\sqrt{2}$ and defining *δ*_*ab*_ = *ϕ*_*a*_ − *ϕ*_*b*_, we get

15 $${m}_{x}=\pm \cos ({\bf{Q}}\cdot {\bf{r}}+{\delta }_{ab}),\quad {m}_{y}=\mp \sin ({\bf{Q}}\cdot {\bf{r}}+{\delta }_{ab}\mathrm{).}$$

Accordingly, the stripes in Fig. 1 indicate a periodic rotation of the electronic spin in and out of the plane, with a wave vector ${\bf{Q}}=4\alpha {\hat{e}}_{x}$ oriented at a 45° angle with respect to the *x*′-axis (i.e., the [110] direction). This is the spin helix pattern^20,27^.

The magnetizations *m*_*x*_ and *m*_*y*_ associated with the states ${{\rm{\Phi }}}_{{\bf{k}}}^{{\boldsymbol{+}}}$ and ${{\rm{\Phi }}}_{{\bf{k}}}^{{\boldsymbol{-}}}$ cancel out; therefore, adding up all occupied spin-helix states below the Fermi energy *E*_*F*_ gives zero. This means that the ground state of the *N*-electron system has no spin texture. The persistent spin helix pattern can be observed if additional quasiparticles are injected at the Fermi level, as shown in Fig. 2a. Such states will have a very long lifetime^20,22,23^.

### *B* ≠ 0: spin-helical single-particle excitations

For a finite magnetic field (Z^*^ > 0), the degeneracy of the two energy branches *E*_+,**k**+**Q**_ and *E*_−,**k**_ is lifted, as shown in Fig. 2b. As a consequence, the spin-helix pattern (15) is not a property of the ground state anymore: instead, the spin helix becomes a nonequilibrium feature.

To see this, consider a single-particle excitation across the Fermi surface, with wave vector transfer **Q**, as illustrated by the thick green arrow in Fig. 2b. First-order perturbation theory tells us that the time-dependent wave function has the form

16 $${{\rm{\Phi }}}_{{\bf{k}}\to {\bf{k}}+{\bf{Q}}}({\bf{r}},t)=\gamma (\begin{array}{c}1\\ 0\end{array}){e}^{i({\bf{k}}+{\bf{Q}})\cdot {\bf{r}}}{e}^{-i({E}_{-,{\bf{k}}}+{Z}^{\ast })t}+(\begin{array}{c}0\\ 1\end{array}){e}^{i{\bf{k}}\cdot {\bf{r}}}{e}^{-i{E}_{-,{\bf{k}}}t},$$

where $|\gamma |\ll 1$ and we made use of Eq. (13). In the ${\mathcal R}$ frame, the *x* and *y* components of the associated magnetization are given by

17 $${m}_{x}(t)=\mathrm{2|}\gamma |\,\cos ({\bf{Q}}\cdot {\bf{r}}-{Z}^{\ast }t+{\varphi }_{\gamma })$$

18 $${m}_{y}(t)=-\mathrm{2|}\gamma |\,\sin ({\bf{Q}}\cdot {\bf{r}}-{Z}^{\ast }t+{\varphi }_{\gamma })\,,$$

where ${\varphi }_{\gamma }$ is the phase of *γ*. This defines a forward propagating spin helix (i.e., a spin-flip wave), with amplitude 2|*γ*|, wave vector **Q**, and group velocity Z^*^Q/*Q*^2^. Single-particle excitations of this type, which are long-lived due to the property (13), were experimentally observed by Walser *et al*.^23,38^ using circularly polarized optical pump pulses and imaging via time-resolved Kerr rotation microscopy. A theoretical study using a drift-diffusion model was recently carried out by Ferreira *et al*.^39^.

So far, our discussion has been for single-particle excitations, i.e., we did not consider collective excitations. In the following Section, we will present a very special case of a collective mode, which we call the spin-helix Larmor mode, which can be viewed as a coherent superposition of the left- and right-propagating single-particle spin helices considered above. As we will see, this gives rise to a collective, standing precessional wave with wave vector $Q$ which is undamped. Propagating collective spin-flip waves and their wave vector dispersions will be considered in Methods.

## Spin-helix Larmor mode

We consider a 2DEG in the presence of a uniform magnetic field ${\bf{B}}=B{\hat{e}}_{z}$, in the reference frame ${\mathcal R}$ of Fig. 1. The many-body Hamiltonian without SOC is

19 $${\hat{H}}_{0}=\sum _{i}(\frac{{\hat{{\bf{p}}}}_{i}^{2}}{2}+\frac{1}{2}Z{\hat{\sigma }}_{z,i})+\frac{1}{2}\sum _{ij}\frac{1}{|{{\bf{r}}}_{i}-{{\bf{r}}}_{j}|}\mathrm{.}$$

We consider the spin-wave operator^9,34,40,41^

20 $${\hat{S}}_{{\bf{q}}}^{+}=\frac{1}{2}\sum _{i}{\hat{\sigma }}_{+,i}{e}^{i{\bf{q}}\cdot {{\bf{r}}}_{i}}$$

(${\hat{\sigma }}_{+}={\hat{\sigma }}_{x}+i{\hat{\sigma }}_{y}$), whose Heisenberg equation of motion, in the absence of SOC, is

21 $$i\frac{d}{dt}{\hat{{S}}}_{{\bf{q}}}^{+}=[{\hat{{S}}}_{{\bf{q}}}^{+},{\hat{H}}_{0}]=-{\omega }_{L}{\hat{{S}}}_{{\bf{q}}}^{+}-{\bf{q}}\cdot {{\bf{J}}}_{{\bf{q}}}^{+}\,\mathrm{.}$$

The second term on the right-hand side of Eq. (21) arises from the commutator of ${\hat{S}}_{{\bf{q}}}^{+}$ with the kinetic part of ${\hat{H}}_{0}$, where

22 $${{\bf{J}}}_{{\bf{q}}}^{+}=\frac{1}{2}\sum _{i}{\hat{\sigma }}_{+,i}{e}^{i{\bf{q}}\cdot {{\bf{r}}}_{i}}({{\bf{p}}}_{i}+{\bf{q}}\mathrm{/2)}$$

is the transverse spin-current operator at wave vector **q**. The Larmor frequency *ω*_*L*_ is equal to the bare Zeeman energy, *ω*_*L*_ = *Z*. For small values of **q**, the equation of motion (21) can be written as

23 $$i\frac{d}{dt}{\hat{{S}}}_{{\bf{q}}}^{+}=-{\omega }_{{\rm{sw}},0}({\bf{q}}){\hat{{S}}}_{{\bf{q}}}^{+}\,\mathrm{.}$$

Here, *ω*_sw,0_(**q**) implicitly contains the coupling between the collective spin waves and the single-particle spin-current dynamics^34^. The real part, $\Re {\omega }_{{\rm{sw}},0}({\bf{q}})={\omega }_{L}+{S}_{{\rm{sw}}}{q}^{2}\mathrm{/2}$, is the spin-wave dispersion, where *S*_sw_ is the spin-wave stiffness (explicit expressions and discussion of *S*_sw_ are provided in the Methods section). The imaginary part of *ω*_sw,0_(**q**) accounts for the damping of the spin wave due to electron-electron interactions^42^.

Now let us include SOC. The SOC Hamiltonian for the spin-helix case is given by [see Eq. (9)]

24 $${\hat{H}}_{{\rm{SOC}}}=-\frac{1}{2}\sum _{i}{\bf{Q}}\cdot {{\bf{p}}}_{i}{\hat{\sigma }}_{z,i},$$

and the total Hamiltonian of the system is $\hat{H}={\hat{H}}_{0}+{\hat{H}}_{{\rm{SOC}}}$. The equation of motion for ${\hat{S}}_{{\bf{q}}}^{+}$ in the presence of SOC is given by

25 $$i\frac{d}{dt}{\hat{S}}_{{\bf{q}}}^{+}=[{\hat{S}}_{{\bf{q}}}^{+},{\hat{H}}_{0}+{\hat{H}}_{{\rm{SOC}}}]=-{\omega }_{L}{\hat{S}}_{{\bf{q}}}^{+}-({\bf{q}}-{\bf{Q}})\cdot {{\bf{J}}}_{{\bf{q}}}^{+}\mathrm{.}$$

We now show that the SOC contribution can be transformed away. We introduce the SU(2) unitary transformation $\hat{U}=\exp [-i{\sum }_{i}{\bf{Q}}\cdot {{\bf{r}}}_{i}{\hat{\sigma }}_{z,i}\mathrm{/2]}$, which leads to

26 $$\hat{U}{\hat{S}}_{{\bf{q}}}^{+}{\hat{U}}^{\dagger }={\hat{S}}_{{\bf{q}}-{\bf{Q}}}^{+},\quad \hat{U}{\hat{J}}_{{\bf{q}}}^{+}{\hat{U}}^{\dagger }={\hat{J}}_{{\bf{q}}-{\bf{Q}}}^{+}\mathrm{.}$$

In other words, $\hat{U}$ causes a boost of the wave vector argument of the spinwave and the spin-current operators, **q** → **q** − **Q**, and transforms the momentum operator of the *i*th electron into $\hat{U}{{\bf{p}}}_{i}{\hat{U}}^{\dagger }={{\bf{p}}}_{i}+{\bf{Q}}{\hat{\sigma }}_{z,i}\mathrm{/2}$. On the other hand, $\hat{U}$ leaves the Coulomb and the Zeeman parts of ${\hat{H}}_{0}$ unchanged. Thus, we obtain

27 $$\hat{U}\hat{H}{\hat{U}}^{\dagger }={\hat{H}}_{0}-\frac{{Q}^{2}}{8}{\hat{\sigma }}_{0}\mathrm{.}$$

Since ${{Q}}^{2}{\hat{\sigma }}_{0}\mathrm{/8}$ commutes with ${\hat{S}}_{{\bf{q}}-{\bf{Q}}}^{+}$, the transformed equation of motion (25) becomes

28 $$i\frac{d}{dt}{\hat{S}}_{{\bf{q}}}^{+}={\hat{U}}^{\dagger }[{\hat{S}}_{{\bf{q}}-{\bf{Q}}}^{+},{\hat{H}}_{0}]\hat{U},$$

where

29 $$[{\hat{S}}_{{\bf{q}}-{\bf{Q}}}^{+},{\hat{H}}_{0}]=-{\omega }_{L}{\hat{S}}_{{\bf{q}}-{\bf{Q}}}^{+}-({\bf{q}}-{\bf{Q}})\cdot {{\bf{J}}}_{{\bf{q}}-{\bf{Q}}}^{+}\mathrm{.}$$

For small values of |**q** − **Q**|, this becomes $[{\hat{S}}_{{\bf{q}}-{\bf{Q}}}^{+},{\hat{H}}_{0}]=-{\omega }_{{\rm{sw}}\mathrm{,0}}({\bf{q}}-{\bf{Q}}){\hat{S}}_{{\bf{q}}-{\bf{Q}}}^{+}$. Substituting this into Eq. (28), we find that the spin waves of the system with *α* = *β* are those of the system without SOC (governed only by ${\hat{H}}_{0}$), but where the wave vector is shifted:

30 $${\omega }_{{\rm{sw}}}({\bf{q}})={\omega }_{{\rm{sw}}\mathrm{,0}}({\bf{q}}-{\bf{Q}}\mathrm{).}$$

Let us now consider the important special case **q** = **Q**. It is known^43^ that, in the absence of SOC, the spin-polarized electron system carries out a collective precessional motion with ${\omega }_{{\rm{sw}}\mathrm{,0}}={\omega }_{L}$. The Larmor frequency *ω*_*L*_ is equal to the bare Zeeman energy, *ω*_*L*_ = *Z*. The Larmor mode has infinite lifetime (zero line width), because it can be represented as a superposition of two exact eigenstates of the system Hamiltonian ${\hat{H}}_{0}$: the many-body ground state |0〉_0_ (the subscript 0 indicates absence of SOC), with ground-state energy ${E}_{0}$, and the many-body eigenstate ${\hat{S}}_{0}^{+}\mathrm{|0}{\rangle }_{0}$, with energy *E*_0_ + *Z*. An important feature of the Larmor’s mode is that it does not carry any spin current in the plane. This is obvious from the equation of motion (21) in the absence of SOC: for the Larmor’s mode which occurs at **q** = 0, no current is induced by the homogenous precession.

Coming back to the case with SOC with the full Hamiltonian $\hat{H}$, we can now formulate the *spin-helix Larmor theorem*. If the spin wave has wave vector **Q**, commensurate with the spin-helix texture, all Coulomb many-body contributions drop out and the frequency is given by the bare Zeeman energy:

31 $${\omega }_{{\rm{sw}}}({\bf{Q}})={\omega }_{{\rm{sw}}\mathrm{,0}}\mathrm{(0)}=Z,$$

which follows directly from Eq. (30). In the presence of SOC, the Larmor’s mode occurs at **q** = **Q** and is not a homogenous mode anymore; however, one still has the property that no spin-current is driven by the precession, since the total current term in Eqs (25) and (29) disappears when **q** = **Q**: this happens because SOC induces spin currents opposite to the spin currents induced by the motion. The spin wave then has vanishing group velocity, ∇_**q**_*ω*_sw_(**q**)|_**q** = **Q**_ = 0, which means that it is a standing wave. All electronic spins precess about their local orientation, given by the spin helix configuration, with the Larmor frequency *ω*_*L*_ = *Z*. The spin-helix Larmor mode is a superposition of two many-body eigenstates of $\hat{H}$: the ground state |0〉 and the state ${\hat{S}}_{{\bf{Q}}}^{+}\mathrm{|0}\rangle$.

**Figure 3:**
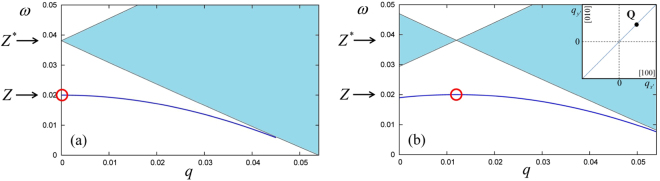
(**a**) Spin-wave dispersion *ω*_sw,0_(**q**) (line) and single-particle spin-flip continuum (shaded area) without SOC. (**b**) Spin-wave dispersion *ω*_sw_(**q**) and single-particle spin-flip continuum, plotted along [110], for *α* = *β* = 0.003 (*Q* = 0.012). The 2DEG parameters are *r*_*s*_ = 2, *ζ* = −0.0762, *Z* = 0.02, and Z^*^ = 0.0381 (all values are in atomic units). The inset shows the position of Larmor’s mode in the wave vector plane (*q*_*x*′_, *q*_*y*′_).

We illustrate the spin-wave dispersions with and without SOC in Fig. 3. The left panel shows *ω*_sw,0_(**q**) (which is independent of the direction of **q**), and the right panel shows *ω*_sw_(**q**) for **q** parallel to ${\bf{Q}}$, i.e., along the [110] direction. For this particular case, *ω*_sw_(**q**) is simply obtained by a horizontal shift by **Q** of *ω*_sw,0_(**q**) (likewise for the particle-hole continua). The spin-wave dispersions plotted in Fig. 3 are obtained from the numerical solution of Eq. (42), see below. The small-wave vector expansion (44) is very close to the exact result.

## Experimental Schemes

Experimental observation of the spin-helix Larmor mode should be possible in specially designed doped magnetic semiconductor quantum well samples where the *α* = *β* condition is met. The spin-flip waves under an in-plane magnetic field can be detected using inelastic light scattering, similar to our earlier work^9,16–18^. The Larmor-type precessional mode about the spin-helix state should then be recognizable by a significant narrowing of the linewidth.

Time-resolved Kerr rotation spectroscopy is another suitable technique to detect collective spin excitations in a 2DEG^44,45^. In the experimental setup by Walser *et al*.^23^ however, only the spin-helical single-particle excitations were observed; at the magnetic field strength of 1 T considered in the experiment, the collective modes were too short-lived to be seen since their separation from the spin-flip continuum was too small and the mode frequency was of the same order as the linewidth.

**Figure 4:**
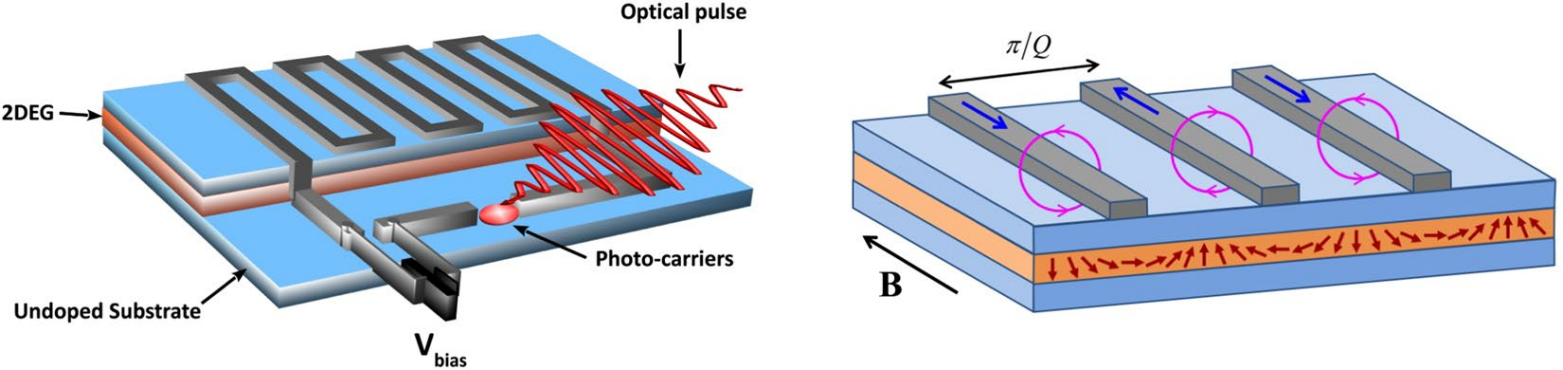
Proposed experimental design for the direct excitation of the spin-helix Larmor mode. Left: photo-conductive antenna on top of the sample, converting an infrared optical pulse into a short current pulse. Right: close-up view of the metal stripes on top of the spin-polarized 2DEG. The currents (blue arrows) are in alternating directions in neighboring stripes; the induced magnetic fields (pink circles) trigger a standing spin wave in the 2DEG.

We also propose a device design which would allow one to excite Larmor’s mode optically and probe it electronically. In the absence of SOC, the Larmor’s mode is usually probed in a paramagnetic resonance setup, by a microwave (mw) magnetic field ${{\bf{b}}}_{{\rm{mw}}}=b\,\cos (\omega t){\hat{e}}_{y}$ applied in the plane perpendicular to the quantizing field ${\bf{B}}=B{\hat{e}}_{z}$. The corresponding coupling Hamiltonian is ${\hat{H}}_{{\rm{mw}}}=-\frac{i}{2}{g}_{e}{\mu }_{B}b\,\cos (\omega t)({\hat{S}}_{0}^{+}-{\hat{S}}_{0}^{-})$. The unitary transformation $\hat{U}$ maps the situation with SOC to the situation without SOC; hence, the coupling Hamiltonian becomes $\hat{U}{\hat{H}}_{{\rm{mw}}}{\hat{U}}^{\dagger }=-\frac{i}{2}{g}_{e}{\mu }_{B}b\,\cos (\omega t)({\hat{S}}_{{\bf{Q}}}^{+}-{\hat{S}}_{{\bf{Q}}}^{-})$. This corresponds to a coupling with a standing helical magnetic field in the plane (*x*, *y*). Such a magnetic field can be generated by a device like the one sketched in Fig. 4. The idea is to deposit metal stripes on top of the sample, separated by a distance *π*/*Q* (for typical values of $\alpha$, of order ~1 meV Å, this corresponds to a few *μm*). The stripes are aligned parallel to the quantizing magnetic field ${\bf{B}}=B{\hat{e}}_{z}$, perpendicular to the [010] direction, and the spacing of the stripes is commensurate with the standing-wave spin-helix Larmor mode. The metal stripes follow the principle of photo-conductive antennas used in time-domain THz experiments^46^. When an infrared optical pulse hits the sample, the photo-generated carriers in the substrate create a short conductive channel closing the biased circuit of the stripes. A current pulse with frequencies in the THz range will propagate through the stripes in alternating direction. This will, by induction, generate concentric oscillating magnetic fields compatible with the spin-helix pattern as depicted in the right panel of Fig. 4. The magnetic field exerts torques on the spin-polarized 2DEG underneath. If the current pulse spectrum contains the right frequency, *ω* = *ω*_*L*_, this will trigger a helical standing spin wave which will persist after the end of the pulse. Detection of the spin-helix Larmor mode, and measurement of its lifetime, should then be possible via the currents induced in the metal stripes from the stray magnetic fields associated with the standing spin wave. Such a detection technique is common in ferromagnetic magnonics^3,4^.

In our semiconductor test-bed system^9^, we deal with lower spin densities and higher frequencies: we expect stray fields of the order of nT, and rapidly varying, at a rate of about 50–100 GHz, with a lifetime in the 100 ps to ns range. The measurements will therefore push the limits of present-day electronics, but they should be feasible. Moreover, the physics and concepts presented here are applicable to emerging 2D conducting systems with higher doping and strong spin-orbit effects. The above estimate for the lifetime of the spin-helix Larmor mode is based on spin-wave linewidths of the order of 50 *μ*eV which were measured^9^ for quantum wells with *α* ≠ *β*; the corresponding dephasing time (165 ps for a linewidth of 50 *μ*eV) can be viewed as a lower threshold to the lifetime of the Larmor mode, since it includes electronic many-body contributions which drop out if *α* = *β*. The lifetime of the spin-helix Larmor mode is mainly determined by cubic Dresselhaus contributions and disorder.

## Discussion

In this paper, we have considered the spin dynamics in a 2DEG in the presence of SOC, under the very special condition where the Rashba and Dresselhaus coupling strengths are equal (*α* = *β*) and where an in-plane magnetic field is applied perpendicular to the [110] direction. Without this magnetic field, the system sustains persistent spin-helix states which have been widely studied in the literature. The magnetic field lifts the degeneracy that leads to the persistent spin-helix states; it instead leads to single-particle excitations that have the form of propagating spin helices.

The presence of Coulomb interactions causes these single-particle excitations to combine and form collective spin waves, which are robust against any decoherence caused by SOC. We have found that for the 2DEG with *α* = *β* the spin-wave dispersion is the same as for the system without SOC, apart for a rigid wave vector shift by **Q** (the spin-helix wave vector). The case of **q** = **Q** thus produces the special scenario which we have termed the spin-helix Larmor mode, where all many-body effects vanish and the precession frequency is given by the bare Zeeman energy (divided by $\hslash$). This is a new and exact result for electronic many-body systems, which opens up new ways of manipulating and driving electronic spins by optical means.

## Methods

We now discuss the spin-wave dispersions in the quantum well system considered above. We have seen that in the case *α* = *β* the single-particle states (11) are pure up and down spinors; therefore, the longitudinal and transverse spin response channels are decoupled. The transverse spin-density response equation reads

32 $${\overrightarrow{n}}_{T}({\bf{q}},\omega )={\underline{\chi }}_{T}({\bf{q}},\omega )[{\overrightarrow{v}}_{T}({\bf{q}},\omega )+{\underline{f}}_{T}^{{\rm{xc}}}({\bf{q}},\omega ){\overrightarrow{n}}_{T}({\bf{q}},\omega )],$$

where ${\overrightarrow{n}}_{T}=({n}_{\uparrow \downarrow },{n}_{\downarrow \uparrow })$ is the transverse spin-density response, ${\overrightarrow{v}}_{T}=({v}_{\uparrow \downarrow },{v}_{\downarrow \uparrow })$ is an external perturbation, and the noninteracting transverse spin response function and the transverse xc kernel are diagonal 2 × 2 matrices in the frame ${\mathcal R}$^41,47^:

33 $$\begin{array}{c}{\underline{\chi }}_{T}({\bf{q}},\omega )=(\begin{array}{cc}{\chi }_{\uparrow \downarrow ,\uparrow \downarrow }({\bf{q}},\omega ) & 0\\ 0 & {\chi }_{\downarrow \uparrow ,\downarrow \uparrow }({\bf{q}},\omega )\end{array}),\\ {\underline{f}}_{T}^{{\rm{x}}c}({\bf{q}},\omega )=(\begin{array}{cc}{f}_{\uparrow \downarrow ,\uparrow \downarrow }^{{\rm{xc}}}({\bf{q}},\omega ) & 0\\ 0 & {f}_{\downarrow \uparrow ,\downarrow \uparrow }^{{\rm{x}}c}({\bf{q}},\omega )\end{array})\mathrm{.}\end{array}$$

The general form of the individual elements of the noninteracting spin-density-matrix response function for a 2DEG is^33^

34 $$\begin{array}{ccc}{\chi }_{\sigma \sigma ^{\prime} ,\tau \tau ^{\prime} }({\bf{q}},\omega ) & = & -\sum _{pp^{\prime} }^{\pm 1}\int \frac{{d}^{2}k}{{(2\pi )}^{2}}\,\frac{f({E}_{p{\bf{k}}})}{\omega -{E}_{p{\bf{k}}}+{E}_{p^{\prime} {\bf{k}}-{\bf{q}}}+i\eta }\\  &  & \times \,[{\delta }_{p,+1}{\delta }_{\sigma \uparrow }+{\delta }_{p,-1}{\delta }_{\sigma \downarrow }]\,[{\delta }_{p^{\prime} ,+1}{\delta }_{\sigma ^{\prime} \uparrow }+{\delta }_{p^{\prime} ,-1}{\delta }_{\sigma ^{\prime} \downarrow }]\\  &  & \times \,{\delta }_{\sigma \tau }{\delta }_{\sigma ^{\prime} \tau ^{\prime} }+\,\sum _{pp^{\prime} }^{\pm 1}\int \frac{{d}^{2}k}{{(2\pi )}^{2}}\,\frac{f({E}_{pk})}{\omega +{E}_{p{\bf{k}}}-{E}_{p^{\prime} {\bf{k}}+{\bf{q}}}+i\eta }\\  &  & \times \,[{\delta }_{p^{\prime} ,+1}{\delta }_{\sigma \uparrow }+{\delta }_{p^{\prime} ,-1}{\delta }_{\sigma \downarrow }]\\  &  & \times \,[{\delta }_{p,+1}{\delta }_{\sigma ^{\prime} \uparrow }+{\delta }_{p,-1}{\delta }_{\sigma ^{\prime} \downarrow }]{\delta }_{\sigma \tau }{\delta }_{\sigma ^{\prime} \tau ^{\prime} }\,,\end{array}$$

where *σ*, *σ*′, *τ*, *τ*′ are spin indices (↑ or ↓), and *η* is a positive infinitesimal (since we will be considering spin waves outside the particle-hole continuum, we can drop *η*). The Fermi function is given by *f*(*E*_*p***k**_) = *θ*(*E*_*F*_ − *E*_*p***k**_), where *E*_*F*_ is the Fermi energy of a paramagnetic 2DEG in the presence of SOC. It can be shown that ${E}_{F}={E}_{F}^{0}-{\alpha }^{2}-{\beta }^{2}$, where ${E}_{F}^{0}=\pi n$ is the Fermi energy of a 2DEG without SOC, and with 2D electronic density *n*.

We recast Eq. (12) as ${E}_{\pm ,{\bf{k}}}=\pm {Z}^{\ast }\mathrm{/2}+\frac{1}{2}{|{\bf{k}}\mp {\bf{Q}}\mathrm{/2}|}^{2}-2{\alpha }^{2}$. With a change of the integration variable, ${\bf{k}}\to {\bf{k}}\pm {\bf{Q}}\mathrm{/2}$, the noninteracting spin-flip response functions become

35 $$\begin{array}{rcl}{\chi }_{\uparrow \downarrow ,\uparrow \downarrow }({\bf{q}},\omega ) & = & -\int \frac{{d}^{2}k}{{\mathrm{(2}\pi )}^{2}}\,\frac{{f}_{0}(\frac{{k}^{2}}{2}+\frac{{Z}^{\ast }}{2})}{\omega -{Z}^{\ast }-{\bf{k}}\cdot ({\bf{q}}-{\bf{Q}})+|{\bf{q}}-{\bf{Q}}{|}^{2}\mathrm{/2}}\\  &  & +\,\int \frac{{d}^{2}k}{{\mathrm{(2}\pi )}^{2}}\,\frac{{f}_{0}(\frac{{k}^{2}}{2}-\frac{{Z}^{\ast }}{2})}{\omega -{Z}^{\ast }-{\bf{k}}\cdot ({\bf{q}}-{\bf{Q}})-|{\bf{q}}-{\bf{Q}}{|}^{2}\mathrm{/2}}\end{array}$$

36 $$\begin{array}{rcl}{\chi }_{\downarrow \uparrow ,\downarrow \uparrow }(q,\omega ) & = & \int \frac{{d}^{2}k}{{\mathrm{(2}\pi )}^{2}}\,\frac{{f}_{0}(\frac{{k}^{2}}{2}+\frac{{Z}^{\ast }}{2})}{\omega +{Z}^{\ast }-{\bf{k}}\cdot ({\bf{q}}+{\bf{Q}})-|{\bf{q}}+{\bf{Q}}{|}^{2}\mathrm{/2}}\\  &  & -\int \frac{{d}^{2}k}{{\mathrm{(2}\pi )}^{2}}\,\frac{{f}_{0}(\frac{{k}^{2}}{2}-\frac{{Z}^{\ast }}{2})}{\omega +{Z}^{\ast }-{\bf{k}}\cdot ({\bf{q}}+{\bf{Q}})+|{\bf{q}}+{\bf{Q}}{|}^{2}\mathrm{/2}}\,,\end{array}$$

where the Fermi function *f*_0_ indicates that *E*_*F*_ has been replaced by ${E}_{F}^{0}$.

Looking at Eqs (35) and (36) we immediately see that the spin-flip response functions of the system with SOC can be expressed in terms of the corresponding functions without SOC (denoted by the superscript 0):

37 $${\chi }_{\uparrow \downarrow ,\uparrow \downarrow }({\bf{q}},\omega )={\chi }_{\uparrow \downarrow ,\uparrow \downarrow }^{0}({\bf{q}}-{\bf{Q}},\omega )$$

38 $${\chi }_{\downarrow \uparrow ,\downarrow \uparrow }({\bf{q}},\omega )={\chi }_{\downarrow \uparrow ,\downarrow \uparrow }^{0}({\bf{q}}+{\bf{Q}},\omega \mathrm{).}$$

This simple result only holds for the special case *α* = *β*. In TDDFT, the spin-flip linear-response xc kernel of the 2DEG is given in the adiabatic local-density approximation (ALDA) by^33^

39 $${f}_{\uparrow \downarrow ,\uparrow \downarrow }^{{\rm{xc}}}={f}_{\downarrow \uparrow ,\downarrow \uparrow }^{{\rm{xc}}}\equiv {K}_{{\rm{xc}}}=\frac{2}{n\zeta }\frac{\partial {e}_{{\rm{xc}}}}{\partial \zeta }\mathrm{.}$$

The spin-flip wave dispersion now follows from Eq. (32) by setting ${\overrightarrow{v}}_{T}$ to zero and finding the eigenmodes, which leads to

40 $$[{K}_{{\rm{xc}}}{\chi }_{\uparrow \downarrow ,\uparrow \downarrow }^{0}({\bf{q}}-{\bf{Q}},{\omega }_{{\rm{sw}}})-\mathrm{1][}{K}_{{\rm{xc}}}{\chi }_{\downarrow \uparrow ,\downarrow \uparrow }^{0}({\bf{q}}+{\bf{Q}},{\omega }_{{\rm{sw}}})-\mathrm{1]}=0.$$

Upon closer inspection of Eqs (35) and (36), we find

41 $${\chi }_{\downarrow \uparrow ,\downarrow \uparrow }^{0}({\bf{q}}+{\bf{Q}},{\omega }_{{\rm{sw}}})={\chi }_{\uparrow \downarrow ,\uparrow \downarrow }^{0}({\bf{q}}-{\bf{Q}},-{\omega }_{{\rm{sw}}}\mathrm{).}$$

We are only interested in positive frequencies, so the spin-flip wave dispersion is obtained from Eq. (40) as the implicit solution *ω*_sw_(**q**) of

42 $${\chi }_{\uparrow \downarrow ,\uparrow \downarrow }^{0}({\bf{q}}-{\bf{Q}},{\omega }_{{\rm{sw}}})={K}_{{\rm{xc}}}^{-1}\mathrm{.}$$

The spin-wave dispersion can be analytically determined for small wave vectors. To second order, we find

43 $${\omega }_{{\rm{sw}}}({\bf{q}})={Z}^{\ast }(1+\frac{{K}_{{\rm{xc}}}}{2\pi })+\frac{{E}_{F}^{0}|{\bf{q}}-{\bf{Q}}{|}^{2}}{{Z}^{\ast }}(1+\frac{2\pi }{{K}_{{\rm{xc}}}})\mathrm{.}$$

This can be rewritten as

44 $${\omega }_{{\rm{sw}}}({\bf{q}})=Z+\frac{1}{2}{S}_{{\rm{sw}}}|{\bf{q}}-{\bf{Q}}{|}^{2},$$

which corresponds to spin waves propagating with group velocity **v**_**g**_(**q**) = **S**_sw_(**q** − **Q**), where the spin-wave stiffness of the 2DEG is

45 $${S}_{{\rm{sw}}}=\frac{2{E}_{F}^{0}}{{Z}^{\ast }}(1+\frac{2\pi }{{K}_{{\rm{xc}}}})\mathrm{.}$$

Since ${K}_{{\rm{xc}}}={Z}_{{\rm{xc}}}/(n\zeta )$ and *ζ* = −Z^*^/(2*πn*), this can also be written as

46 $${S}_{{\rm{sw}}}=-\frac{2{E}_{F}^{0}}{{Z}^{\ast }}(\frac{Z}{{Z}^{\ast }-Z})\mathrm{.}$$

**Figure 5:**
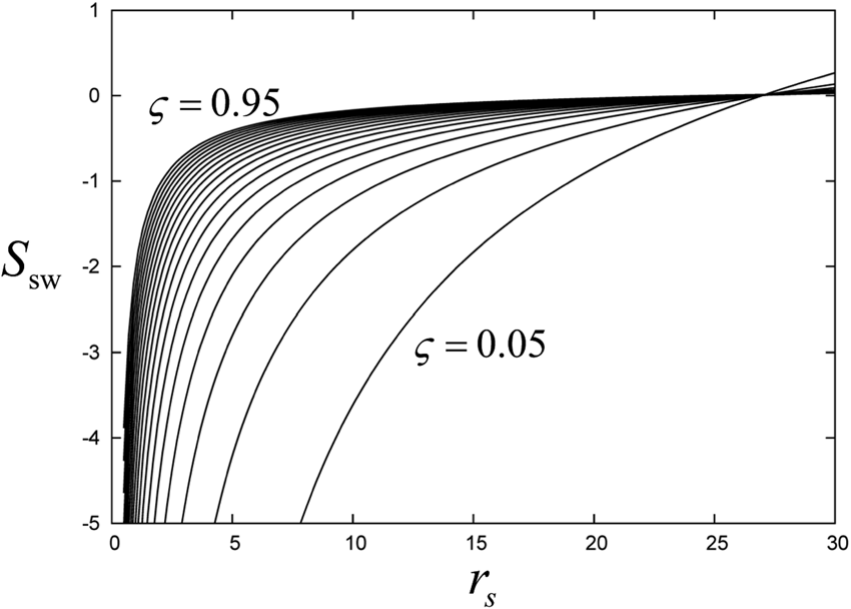
Spin-wave stiffness of the 2DEG, obtained with the ALDA, for various values of the spin polarization *ζ* between 0.05 and 0.95.

We plot the ALDA spin-wave stiffness for various values of the spin polarization *ζ* and as a function of the 2D Wigner-Seitz radius *r*_*s*_ in Fig. 5. The stiffness has negative values for all practically relevant values of *r*_*s*_, and crosses over to *S*_sw_ > 0 for very low densities (at *r*_*s*_ = 26.96 for *ζ* = 0.05 and at *r*_*s*_ = 24.49 for *ζ* = 0.95). In practice, one is interested in studying collective modes in the typical metallic range of *r*_*s*_ between about 2 and 6; this is the intermediate coupling regime where the Coulomb energy (which scales as ${r}_{s}^{-1}$) is comparable to the kinetic energy (which scales as ${r}_{s}^{-2}$), but which can still be regarded as weakly correlated. As an example, in the quantum well system considered in ref.^37^ the spin-wave stiffness was *S*_sw_ = −27.6, for *r*_*s*_ = 2.2 and *ζ* = 0.053.

### Data availability

All data generated or analysed during this study are included in this article.
